# Editorial: The self-renewal and reprogramming of cancer stem cells and their crosstalk with the immune microenvironment 

**DOI:** 10.3389/fcell.2022.1024761

**Published:** 2022-11-11

**Authors:** Chong Li, Jayanth Kosagisharaf Rao, Lakshmi Sowmya Emani, Rao Jagannatha Kosagisharaf, Muralidhar L. Hegde

**Affiliations:** ^1^ Institute of Biophysics, Chinese Academy of Sciences, Beijing, China; ^2^ Zhongke Jianlan Medical Research Institute, Beijing, China; ^3^ Graduate Medical Student, University College of Dublin Medical School, Dublin, Ireland; ^4^ Department of Biotechnology, Koneru Lakshmaiah Education Foundation, Vaddeswaram, India; ^5^ National Science System (SNI), SENACYT, Clayton, City of Knowledge, Panama; ^6^ Division of DNA Repair Research, Center for Neuroregeneration, Department of Neurosurgery, Houston Methodist Research Institute, Houston, TX, United States; ^7^ Department of Neurology, Weill Medical College of Cornell University, New York, NY, United States

**Keywords:** cancer, drug discovery, reprogramming, mRNA, prevention, mechanism

Cancer is an umbrella of complex diseases, where cells from any organ of the body will start growing uncontrollably leading to cell dysfunction. This uncontrolled growth leads to the spread of these cancer cells to adjacent tissues like muscles, bones, and other distal organs in the body. This process is defined as metastasis, which leads to the development of cancerous cells affecting the function of the invaded tissue site. The process and degree of metastasis warrant poor prognosis and eventually lead to the death of the individual. In men, the predominant cancer types include liver, prostate, gastro-intestinal, and colorectal, but in women, the major cancer types are the breast, cervical, and colorectal. Cancer is the second most common cause of death globally (WHO, 2022a). The WHO counts around 10 million deaths caused by a form of cancer in 2020. The most prevalent cases of cancer seen in 2020 (in millions) are 2.26-breast cancer, 2.21-lung cancer, 1.93-colorectal cancer, 1.41-prostate cancer, 1.20-non-melanoma skin cancer, and 1.09-stomach cancer. The most common cancers causing mortality in 2020 (in millions) are 1.80-lung cancer, 0.91-colorectal cancer, 0.83-liver cancer, 0.76-stomach cancer, and 0.68-breast cancer. Further, the WHO also reported that every year 400,000 children develop cancer, and the etiology of this pediatric cancer is still a debate. They have also estimated that cancer related to cervical tissue is predominant in women in 23 countries, and the understanding of the cancer epidemiology from country to country is still complex (WHO, 2022b).

Cancer’s burden on the health systems of middle and low-income countries is increasing every year, thus affecting the quality of healthcare around the world. Though cancer is an immense emerging threat, it can still be prevented from reaching advanced stages. The WHO estimates that 30%–50% of cancer deaths can be prevented by exercising caution against major risk factors and early screening strategies for cancer. Evidence-based preventative strategies can be formulated for the early detection of cancer, leading to a lower burden of the disease on both the individual and help healthcare system (WHO, 2022a).

The biochemical understanding of normal cells and their transformation into cancer cells is critical in cancer biology and is still a challenging opportunity for researchers. Cancer stem cells (CSCs) are the complex sub-population of tumor cells in the human body, and have the ability to program themselves into differentiation and de-differentiation pathways. These metabolic characters contribute to their ability to form tumor-initiating pathways like tumor metastasis, relapsing, and the development of chemo-resistance. The cellular microenvironment provides a congenial system for CSCs in the body to retain the ability to express self-renewal and differentiation capacity. Also, it is interesting to mention that the CSCs have the ability to adapt to the cellular tumor microenvironment and modify themselves epigenetically, and immunologically, leading to the metabolic diversion to suit and survive in this new tumor microenvironment. The changes in the tumor environment induce the chronic inflammation condition in the CSC microenvironment favoring proliferation ability.

A large number of studies in recent times highlighted the complexity in the cellular cross-talk of CSCs among immunomodulators; self-renewal capacity, cell survivability, reprogramming ability, etc, and all these biochemical pathways potentially favor tumorigenicity, metastasis, and development of resistance to chemotherapy, etc. But the clear understanding of the above complexity of CSCs adapting to these cellular microenvironmental changes leading to tumor formations is still not clearly understood hence these are crucial steps in future Cancer Research. Further, the metabolic adaptation leading to chemo-resistance is a topic of great interest for basic as well as clinical researchers.

The goal of this Research Topic on “*The Self-Renewal and Reprogramming of Cancer Stem Cells and Their Crosstalk with the Immune Microenvironment*”, in the lead journal Frontiers in Cell and Developmental Biology is to bring together research contributions that help us to understand and elucidate the current findings on the pathways involved in self-renewal and metabolic programming of CSCs in the tumor microenvironment in the human body leading to cancer development. We focused on the topics relevant to CSCs and the role of cellular self-renewal, the discovery of novel subpopulations of CSCs, novel biomarkers of CSCs, metabolic adaptation to the tumor microenvironment, immunological challenges, the complex issues associated with CSCs to adapt to this new microenvironment, to discover novel metabolic pathways involved in cancer formation, and finally to discover the mechanisms on why and how cancer cells develop resistance against drugs.

The following six original articles are accepted and published under the Research Topic: a) High Expression MicroRNA-206 Inhibits the Growth of Tumor Cells in Human Malignant Fibrous Histiocytoma by Li et al.; The major focus of this paper is to identify the role of dysregulation of miRNAs in MFH, CSCs and they discovered that Hsa-miR-206 has a pivotal role in modulating MFH CSC properties and may represent as biomarker and future therapeutic target; b) miR-92a-3p Promoted EMT *via* Targeting LATS1 in Cervical Cancer Stem Cells by Liu et al.; the article helps in exploring the role of miR-92a-3p in cervical cancer (CC) and they found that cyclin E. miR-92a-3p promoted the malignant activity of CC stem cells through LATS1; c) Deficient or R273H and R248W Mutations of p53 Promote Chemoresistance to 5-FU *via* TCF21/CD44 Axis-Mediated Enhanced Stemness in Colorectal Carcinoma, Gao et al.; the investigators tried to unravel the role of specific mutant p53 which enhances carcinogenesis and chemoresistance. Their findings gave the first insight that the chemoresistance pathway in colorectal carcinoma is directed by mutants in p53/TCF21/CD44; d) Multi-Omics Characterization of Tumor Microenvironment Heterogeneity and Immunotherapy Resistance Through Cell States–Based Subtyping in Bladder Cancer, Hu et al.; they found that PD-1 may influence objective response rate (ORR) in BC and these findings open up new research directions; e) Identification and Development of Inflammatory Response–Related Genes Signature Associated With Prognosis Evaluation and Immune Status of Bladder Cancer, Zheng et al.; gave clear evidence that a novel prognostic signature involving seven IRGs in modulating the inter-relationship of immune features in BLCA and the associated risk scores; f) PRC1 and RACGAP1 are Diagnostic Biomarkers of Early HCC and PRC1 Drives Self-Renewal of Liver Cancer Stem Cells, Liao et al.; they focused on screening of PRC1 and RACGAP1 as novel and predictive biomarkers for early HCC detection. Further, two review articles are published in this special Research Topic, focused on g) Epigenetic Signaling of Cancer Stem Cells during Inflammation, Liu et al.; and h) The Origin and Evolution of Bladder Cancer Stem Cell, Tan et al., highlighted the importance of modulations in epigenetics and the pattern of evolution in CSCs during tumor progression.

All these articles opened up new research directions to further understand the novel DNA damage pathways, the role of micro RNAs in regulating or failing to regulate gene expression in cancer cells, new pathways which block cell differentiation, the regulation of miRNA pool levels in cancer cells, miRNA as transcription modulators to control cell proliferation and modulating genomic stability in cancer cells. All these findings open up new research avenues with translational applications which are a future hope for cancer treatment. A critical understanding of the above factors is essential to map the complex issues in cancer biology and near future, there is a high possibility to develop novel metabolic pathways targeted drugs, with fewer side effects.
